# American Heart Month — February 2014

**Published:** 2014-02-14

February is American Heart Month. Cardiovascular disease (CVD), including heart disease, stroke, and high blood pressure, is the leading cause of death among women and men in the United States as well as a leading cause of disability (1). CVD costs the United States approximately $300 billion each year, including the cost of health-care services, medications, and lost productivity from premature death (1).

CVD does not affect all persons in the same way. Factors such as age, race, ethnicity, and sex can affect a person’s risk for heart disease. Regardless, CVD and risk factors are largely preventable with changes in health habits, community changes to create healthier living spaces, and improvement of quality of care (2).

In observance of American Heart Month, CDC has published an online feature article focusing on CVD (available at http://www.cdc.gov/features/heartmonth ), which includes information to help persons take control of their heart health using the “ABCS”: A) take aspirin as directed by your health-care provider; B) control your blood pressure; C) manage your cholesterol; and S) don’t smoke.

Additional information about CVD and heart health is available this month and throughout the year at http://millionhearts.hhs.gov/index.html.
